# A dibenzofuran derivative: 2-(pentyloxy)dibenzo[*b,d*]furan

**DOI:** 10.1107/S2414314618013068

**Published:** 2018-09

**Authors:** Navneet Goyal, James P. Donahue, Camilla Do, Timothy Perry, Kyla Bongay-Williams, Maryam Foroozesh

**Affiliations:** a Department of Chemistry, Xavier University of Louisiana, 1 Drexel Dr., New Orleans, Louisiana 70125, USA; b Department of Chemistry, Tulane University, 6400 Freret Street, New Orleans, Louisiana 70118-5698, USA

**Keywords:** crystal structure, dibenzofuran, π-π stacking interactions, hydrophobic interactions, P450 inhibitor

## Abstract

The title compound, C_17_H_18_O_2_, crystallizes in two-dimensional sheets, in which the 2-(pentyloxy)dibenzo[*b*,*d*]furan molecules are arranged in a head-to-head and tail-to-tail fashion that enables hydrophobic interactions between fully extended 2-pentoxy chains and π-π stacking between dibenzofuran rings in adjacent rings. Nearest intermolecular π-π stacking contacts are 3.3731 (12) Å. The molecule is nearly planar with an r.m.s. deviation of 0.0803 Å from the mean plane defined by the nineteen non-hydrogen atoms.

## Structure description

Natural products and their structurally diverse derivatives play a major role in drug discovery and development (Cragg *et al.,* 1997). Derivatives of dibenzofuran, a three-ring fused system, have shown interesting biological activities as therapeutics for diseases such as cancer, thombosis, tuberculosis, *etc* (Yurtasş *et al.,* 2016; Kantevari *et al.,* 2011; Chiranjeevi *et al.,* 2013). Our lab is studying the design and synthesis of new inhibitors of P450 enzymes, which are a superfamily of heme proteins involved in the metabolism and detoxification of endogenous and exogenous compounds (Sridhar *et al.,* 2017). P450s are involved in the bioactivation of certain procarcinogens leading to the production of carcinogenic species. The development of potent and selective P450 enzyme inhibitors has attracted considerable attention over the years and has become an important cancer prevention target (Alexander *et al.,* 1995; Foroozesh *et al.,* 1997; Sridhar *et al.,* 2017; Foroozesh *et al.,* 2013).

Previous studies in our laboratory have shown that P450 1A1 accommodates linear polycyclic aromatic molecules while P450 1A2 prefers triangle-shaped molecules. [The 1A1 nomenclature designates enzymes belonging to family 1, subfamily A, polypeptide 1, as encoded by the CYP1A1 gene; the 1A2 notation is similarly defined.] These substrate preferences have led to the design of several triangle-shaped carbazole derivatives in an attempt to synthesize potentially selective inhibitors for P450 1A2 over P450 1A1. We are also interested in synthesizing molecules that have fused-ring systems such as dibenzofuran in our pursuit of active P450 inhibitors.

**Figure 1:**
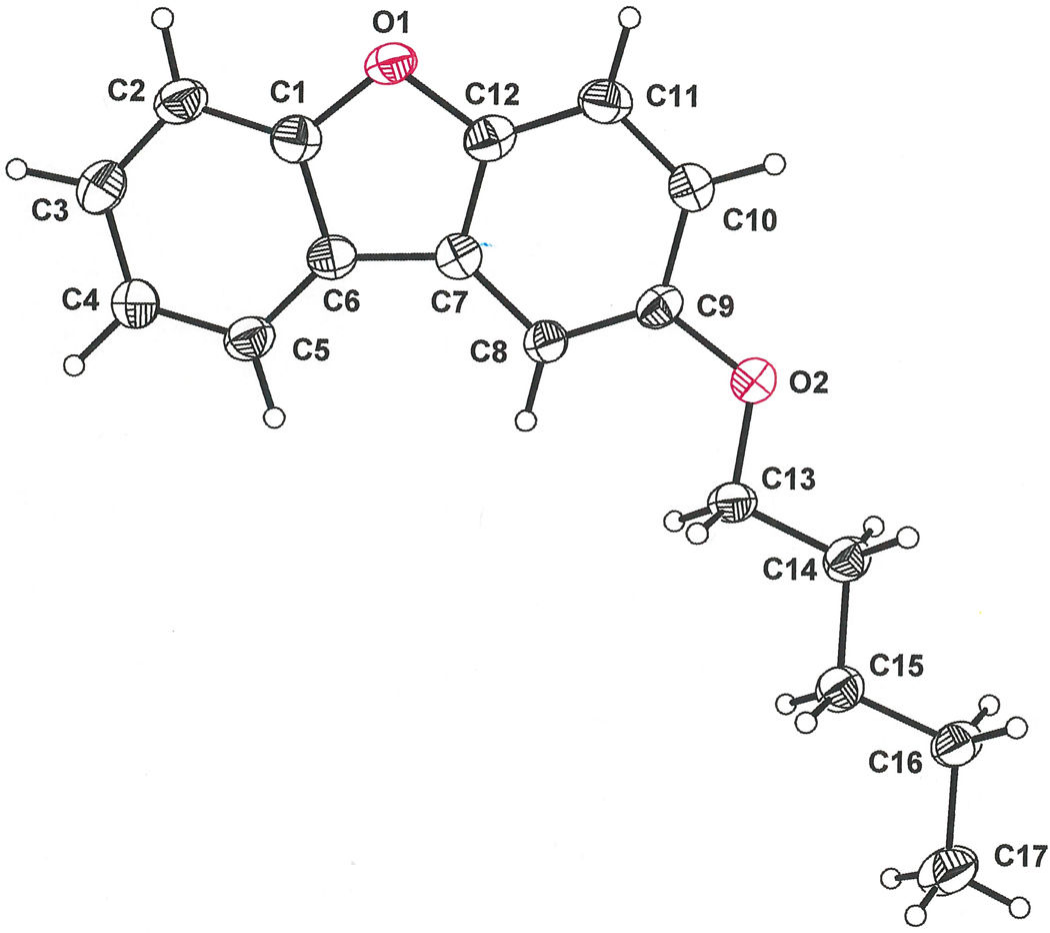
The title molecule with the atom-labeling scheme and 50% probability ellipsoids.

**Figure 2:**
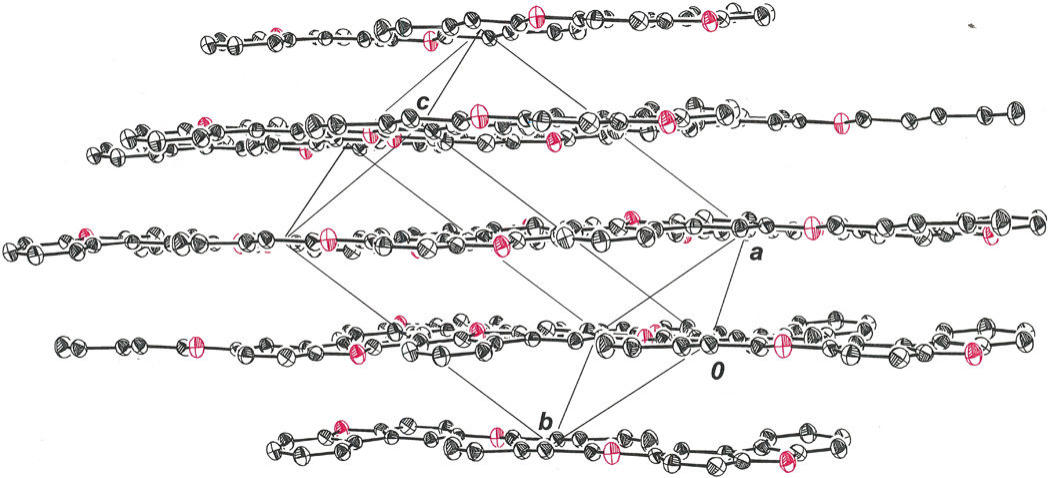
Packing of molecules of 2-(pentyloxy)dibenzo[*b*,*d*]furan within the unit cell. Displacement ellipsoids are shown at the 50% probability level.

**Figure 3:**
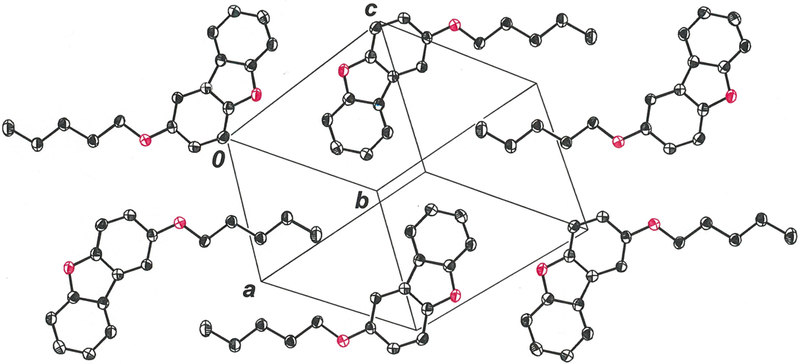
Molecules of 2-(pentyloxy)dibenzo[*b*,*d*]furan within a sheet, showing the tail-to-tail arrangement of pentyloxy substituents. Displacement ellipsoids are shown at the 50% probability level.

**Figure 4:**
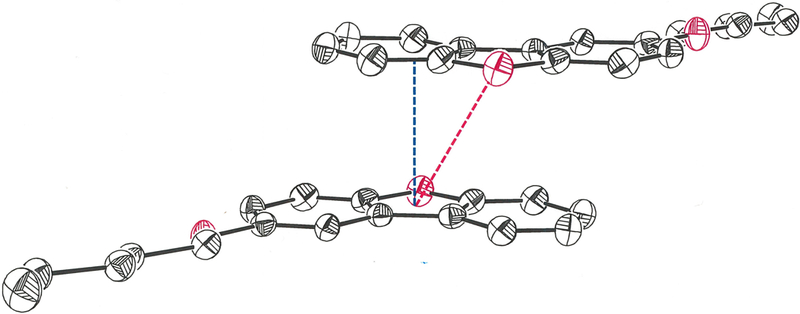
Molecules of 2-(pentyloxy)dibenzo[*b*,*d*]furan in adjacent sheets. The furan ring centroid-to-centroid distance is depicted with the red dashed line, while the separation along a perpendicular from a furan ring centroid is shown in blue. Displacement ellipsoids are shown at the 50% probability level.

In the crystalline state, the 2-pentyloxy substituent in the title compound occurs in a fully extended, linear conformation in which it is nearly coplanar with the dibenzofuran ring system (Fig. 1). The r.m.s. deviation from the mean plane defined by the nineteen non-hydrogen atoms is 0.0803 Å, with the largest departure being seen for O2 at 0.178 (2) Å. The distinctive feature of the molecular packing is an arrangement of the molecules into two-dimensional sheets that are neither parallel nor orthogonal to any cell axis or face (Fig. 2). Within these sheets, adjacent molecules are juxtaposed in a head-to-head and tail-to-tail fashion such that pseudo twofold rotational symmetry relates them (Fig. 3). An apparent consequence of this pattern is the creation of interchain hydrophobic interactions between pentyloxy groups, which likely assist in supporting their fully extended, linear disposition. Molecules of 2-(pentyloxy)dibenzo[*b*,*d*]furan in adjacent sheets enjoy π-π stacking interactions that slightly offset the centroid of the furan ring of one molecule above that of a neighboring molecule such that the centroid-to-centroid distance is 4.070 (3) Å (Fig. 4, red line). The length of the perpendicular segment between adjacent furan rings, defined with one point as a furan centroid, is 3.3731 (12) Å (Fig. 4, blue line). These separations are comparable to the 3.72–3.76 Å distances between molecules in the structure of dibenzofuran itself (Dideberg *et al.,* 1972).

## Synthesis and crystallization

The starting material, 2-hydroxydibenzofuran (0.10 g, 0.54 mmol), was dissolved in 10 mL of acetone under an N_2_ atmosphere. To this solution, solid potassium carbonate was then added (0.3 g, 4 eq.). The reaction mixture was stirred at room temperature for 30 minutes before the dropwise addition of pentyl bromide (0.10 mL, 0.59 mmol). Mild heating to 45°C was applied for 4–5 h, during which time the reaction progress was monitored using thin layer chromatography. The reaction mixture was cooled to room temperature, vacuum filtered, and then taken to dryness under reduced pressure. The crude solid residual was purified by flash chromatography on a silica column eluted with 10:90 EtOAc:hexanes to afford the target compound as a white solid. Crystals were obtained by slow cooling of a warm solution in ethyl acetate:hexanes (2:1, *v:v*). Yield: 0.35 g, 85%. *R*_f_*:* 0.80 (10:90 EtOAc:hexanes, UV). ^1^H NMR (300 MHz, (δ, ppm in CDCl_3_): 7.94 (*d*, *J* = 8.2 Hz, 1H), 7.59 (*d*, *J* = 8.3 Hz, 1H), 7.52–7.44 (*m*, 3H), 7.39–7.33 (*m*, 1H), 7.11–7.06 (*m*, 1H), 4.09 (*t*, *J* = 6.5 Hz, 2H), 1.89 (*q*, *J* = 7.2 Hz, 2H), 1.41–1.59 (*m*, 4H), 1.00 (*t*, *J* = 7.1 Hz, 3H). ^13^C NMR (75 MHz, (δ, ppm in CDCl_3_): 156.9, 155.4, 150.8 127.0, 124.6, 124.5, 122.4, 120.5, 115.7, 112.0, 104.6, 69.0, 29.1, 28.3, 22.5, 14.1. GC–MS: 254, 183.

## Refinement

Crystal data, data collection and structure refinement details are summarized in Table 1.

## Supplementary Material

Sup

## Figures and Tables

**Figure:**
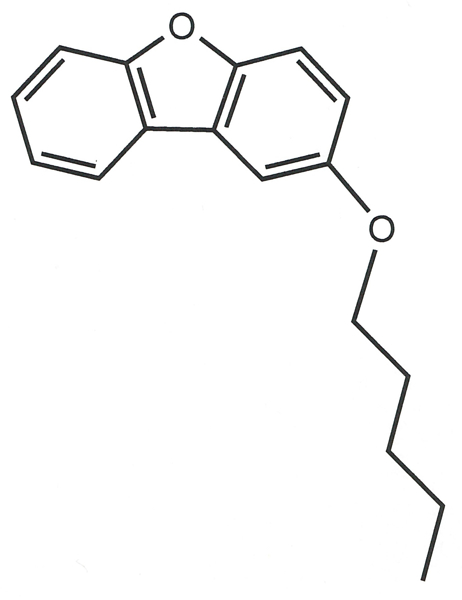
Chemical scheme

**Table 1:** Experimental details

Crystal data	
Chemical formula	C_17_H_18_O_2_
*M*_r_	254.31
Crystal system, space group	Triclinic, *P*$\bar{1}$
Temperature (K)	150
*a*, *b*, *c* (Å)	7.841 (4), 8.203 (4), 11.080 (6)
α, β, γ (°)	79.131 (7), 85.616 (6), 74.077 (6)
*V* (Å^3^)	672.8 (6)
*Z*	2
Radiation type	Mo *K*α
μ (mm^−1^)	0.08
Crystal size (mm)	0.39 × 0.23 × 0.15

Data collection	
Diffractometer	Bruker *SMART APEX* CCD
Absorption correction	Multi-scan (*SADABS*; Krause *et al.,* 2015)
*T*_min_, *T*_max_	0.790, 0.987
No. of measured, independent and observed [*I* > 2σ(*I*)] reflections	4601,1806, 1345
*R*_int_	0.029
θ_max_ (°)	22.7
(sin θ/λ)_max_ (Å^−1^)	0.544

Refinement	
*R*[*F*^2^ > 2σ(*F*^2^)], *wR*(*F*^2^), *S*	0.052, 0.152, 1.11
No. of reflections	1806
No. of parameters	244
H-atom treatment	All H-atom parameters refined
Δρ_max_, Δρ_min_ (e Å^−3^)	0.23, −0.24

Computer programs: *APEX3* and *SAINT* (Bruker, 2016), *SHELXT* (Sheldrick, 2015*a*), *SHELXL2018/1* (Sheldrick, 2015*b*) and *SHELXTL* (Sheldrick, 2008).

## Structural data

Crystal structure: contains datablock I. DOI: https://doi.org//10.1107/S2414314618013068/bx4012sup1.cif

Structure factors: contains datablock I. DOI: https://doi.org//10.1107/S2414314618013068/bx4012Isup2.hkl

### Crystal data

**Table:**

C_17_H_18_O_2_	*Z* = 2
*M*_*r*_ = 254.31	*F*(000) = 272
Triclinic, *P*$\bar{1}$	$D_{\times }$ = 1.255 Mg m^−3^
*a* = 7.841 (4) Å	Mo *K*α radiation, λ = 0.71073 Å
*b* = 8.203 (4) Å	Cell parameters from 2845 reflections
*c* = 11.080 (6) Å	θ = 2.6–22.7°
α = 79.131 (7)°	μ = 0.08 mm^−^
β = 85.616 (6)°	*T* = 150 K
γ = 74.077 (6)°	Block, colorless
*V* = 672.8 (6) Å^3^	0.39 × 0.23 × 0.15 mm

### Data collection

**Table:**

Bruker SMART APEX CCD diffractometer	1806 independent reflections
Radiation source: fine-focus sealed tube	1345 reflections with *I* > 2σ(*I*)
Graphite monochromator	*R*_int_ = 0.029
Detector resolution: 8.3333 pixels mm^−1^	θ_max_ = 22.7°, θ_min_ = 1.9°
φ and ω scans	*h* = −8→8
Absorption correction: multi-scan (*SADABS*; Krause *et al.*, 2015)	*k* = −8→8
*T*_min_ = 0.790, *T*_max_ = 0.987	*l* = −12→11
4601 measured reflections	

### Refinement

**Table:**

Refinement on *F*^2^	Primary atom site location: structure-invariant direct methods
Least-squares matrix: full	Secondary atom site location: difference Fourier map
*R*[*F*^2^ > 2σ(*F*^2^)] = 0.052	Hydrogen site location: difference Fourier map
*wR*(*F*^2^) = 0.152	All H-atom parameters refined
*S* = 1.11	*w* = 1/[σ^2^(*F*_o_^2^) + (0.0701*P*)^2^ + 0.5606*P*] where *P* = (*F*_o_^2^ + 2*F*_c_^2^)/3
1806 reflections	(Δ/σ)_max_ < 0.001
244 parameters	Δϱ_max_ = 0.23 e Å^−3^
0 restraints	Δϱ_min_ = −0.24 e Å^−3^

### Special details

#### Experimental.

The diffraction data were obtained from 3 sets of 400 frames, each of width 0.5° in ω, collected at ϕ = 0.00, 90.00 and 180.00° and 2 sets of 800 frames, each of width 0.45° in ϕ, collected at ω = –30.00 and 210.00°. The scan time was 30 sec/frame.

#### Geometry.

All esds (except the esd in the dihedral angle between two l.s. planes) are estimated using the full covariance matrix. The cell esds are taken into account individually in the estimation of esds in distances, angles and torsion angles; correlations between esds in cell parameters are only used when they are defined by crystal symmetry. An approximate (isotropic) treatment of cell esds is used for estimating esds involving l.s. planes.

### Fractional atomic coordinates and isotropic or equivalent isotropic displacement parameters (Å^2^)

**Table:**

	*x*	*y*	*z*	*U*_iso_*/*U*_eq_
O1	0.0899 (2)	0.7991 (2)	0.40165 (18)	0.0332 (6)
O2	0.2926 (2)	0.3931 (3)	0.04899 (18)	0.0349 (6)
C1	−0.0704 (4)	0.7661 (4)	0.4407 (3)	0.0306 (7)
C2	−0.1831 (4)	0.8459 (4)	0.5259 (3)	0.0345 (8)
H2	−0.152 (3)	0.931 (3)	0.559 (2)	0.019 (7)*
C3	−0.3401 (4)	0.8019 (4)	0.5513 (3)	0.0369 (8)
H3	−0.433 (5)	0.856 (4)	0.611 (3)	0.056 (10)*
C4	−0.3820 (4)	0.6819 (4)	0.4941 (3)	0.0351 (8)
H4	−0.504 (4)	0.650 (4)	0.516 (3)	0.033 (7)*
C5	−0.2659 (4)	0.6033 (4)	0.4094 (3)	0.0326 (8)
H5	−0.290 (4)	0.518 (4)	0.369 (3)	0.033 (8)*
C6	−0.1074 (4)	0.6445 (3)	0.3816 (3)	0.0285 (7)
C7	0.0414 (3)	0.5998 (3)	0.2969 (2)	0.0264 (7)
C8	0.0839 (4)	0.4924 (4)	0.2096 (3)	0.0271 (7)
H8	0.005 (4)	0.427 (4)	0.197 (2)	0.027 (7)*
C9	0.2361 (3)	0.4897 (3)	0.1411 (2)	0.0265 (7)
C10	0.3479 (4)	0.5903 (4)	0.1570 (3)	0.0309 (7)
H10	0.454 (4)	0.588 (4)	0.106 (3)	0.033 (8)*
C11	0.3068 (4)	0.6953 (4)	0.2441 (3)	0.0332 (8)
H11	0.387 (3)	0.760 (3)	0.255 (2)	0.018 (6)*
C12	0.1539 (4)	0.6982 (4)	0.3117 (3)	0.0297 (7)
C13	0.2041 (4)	0.2662 (4)	0.0379 (3)	0.0293 (7)
H13A	0.208 (3)	0.182 (4)	0.115 (3)	0.029 (7)*
H13B	0.072 (4)	0.319 (3)	0.023 (2)	0.025 (7)*
C14	0.2947 (4)	0.1780 (4)	−0.0661 (3)	0.0311 (7)
H14B	0.289 (3)	0.264 (3)	−0.139 (3)	0.019 (7)*
H14A	0.423 (4)	0.131 (4)	−0.048 (2)	0.030 (7)*
C15	0.2184 (4)	0.0354 (4)	−0.0873 (3)	0.0321 (7)
H15A	0.220 (3)	−0.045 (4)	−0.009 (3)	0.029 (7)*
H15B	0.091 (4)	0.087 (4)	−0.111 (2)	0.031 (7)*
C16	0.3193 (4)	−0.0593 (4)	−0.1866 (3)	0.0344 (8)
H16B	0.327 (4)	0.024 (4)	−0.264 (3)	0.032 (8)*
H16A	0.447 (4)	−0.110 (3)	−0.163 (2)	0.030 (7)*
C17	0.2355 (5)	−0.1938 (5)	−0.2144 (4)	0.0479 (9)
H17A	0.295 (4)	−0.255 (4)	−0.279 (3)	0.040 (9)*
H17C	0.229 (5)	−0.279 (5)	−0.140 (4)	0.068 (12)*
H17B	0.106 (5)	−0.136 (5)	−0.242 (3)	0.074 (12)*

### Atomic displacement parameters (Å^2^)

**Table:**

	*U*^11^	*U*^22^	*U*^33^	*U*^12^	*U*^13^	*U*^23^
O1	0.0338 (12)	0.0302 (12)	0.0416 (13)	−0.0115 (9)	0.0012 (9)	−0.0171 (10)
O2	0.0346 (12)	0.0360 (12)	0.0393 (12)	−0.0138 (9)	0.0081 (9)	−0.0164 (10)
C1	0.0280 (16)	0.0264 (16)	0.0365 (17)	−0.0044 (13)	−0.0030 (13)	−0.0062 (14)
C2	0.0376 (18)	0.0279 (17)	0.0403 (19)	−0.0080 (14)	−0.0004 (14)	−0.0131 (15)
C3	0.0377 (18)	0.0330 (18)	0.0382 (19)	−0.0032 (14)	0.0024 (14)	−0.0125 (15)
C4	0.0325 (17)	0.0360 (18)	0.0385 (18)	−0.0098 (14)	0.0043 (14)	−0.0110 (15)
C5	0.0376 (18)	0.0263 (17)	0.0352 (18)	−0.0086 (14)	−0.0004 (14)	−0.0091 (14)
C6	0.0304 (16)	0.0252 (16)	0.0296 (16)	−0.0063 (12)	−0.0038 (13)	−0.0042 (13)
C7	0.0270 (15)	0.0254 (16)	0.0256 (16)	−0.0056 (12)	−0.0026 (12)	−0.0025 (13)
C8	0.0249 (15)	0.0276 (16)	0.0306 (17)	−0.0072 (13)	−0.0001 (12)	−0.0093 (13)
C9	0.0278 (15)	0.0236 (16)	0.0277 (16)	−0.0027 (12)	−0.0044 (12)	−0.0079 (13)
C10	0.0284 (16)	0.0338 (17)	0.0316 (17)	−0.0101 (13)	0.0027 (14)	−0.0073 (14)
C11	0.0310 (17)	0.0324 (17)	0.0398 (19)	−0.0120 (14)	−0.0043 (14)	−0.0085 (15)
C12	0.0300 (16)	0.0265 (16)	0.0324 (17)	−0.0041 (12)	−0.0063 (13)	−0.0072 (14)
C13	0.0312 (17)	0.0242 (16)	0.0353 (19)	−0.0094 (13)	−0.0015 (13)	−0.0085 (14)
C14	0.0309 (18)	0.0268 (17)	0.0354 (19)	−0.0048 (13)	−0.0017 (13)	−0.0087 (15)
C15	0.0322 (18)	0.0289 (17)	0.0357 (19)	−0.0066 (14)	−0.0017 (14)	−0.0087 (15)
C16	0.0365 (19)	0.0305 (18)	0.038 (2)	−0.0077 (14)	−0.0024 (14)	−0.0128 (16)
C17	0.057 (2)	0.041 (2)	0.053 (2)	−0.0146 (18)	−0.0008 (19)	−0.023 (2)

### Geometric parameters (Å, °)

**Table:**

O1—C1	1.378 (3)	C10—C11	1.375 (4)
O1—C12	1.392 (3)	C10—H10	0.96 (3)
O2—C9	1.382 (3)	C11—C12	1.361 (4)
O2—C13	1.428 (3)	C11—H11	0.96 (3)
C1—C2	1.375 (4)	C13—C14	1.504 (4)
C1—C6	1.394 (4)	C13—H13A	0.99 (3)
C2—C3	1.370 (4)	C13—H13B	1.02 (3)
C2—H2	0.94 (3)	C14—C15	1.514 (4)
C3—C4	1.387 (4)	C14—H14B	0.96 (3)
C3—H3	1.01 (4)	C14—H14A	1.00 (3)
C4—C5	1.378 (4)	C15—C16	1.514 (4)
C4—H4	1.06 (3)	C15—H15A	0.99 (3)
C5—C6	1.373 (4)	C15—H15B	1.00 (3)
C5—H5	0.96 (3)	C16—C17	1.516 (4)
C6—C7	1.451 (4)	C16—H16B	1.00 (3)
C7—C8	1.390 (4)	C16—H16A	1.01 (3)
C7—C12	1.386 (4)	C17—H17A	0.97 (3)
C8—C9	1.362 (4)	C17—H17C	0.99 (4)
C8—H8	0.96 (3)	C17—H17B	1.04 (4)
C9—C10	1.399 (4)		
C1—O1—C12	105.0 (2)	C10—C11—H11	119.0 (15)
C9—O2—C13	118.3 (2)	C11—C12—O1	125.3 (2)
C2—C1—O1	124.3 (3)	C11—C12—C7	122.9 (3)
C2—C1—C6	123.6 (3)	O1—C12—C7	111.8 (2)
O1—C1—C6	112.1 (2)	O2—C13—C14	107.2 (2)
C3—C2—C1	116.7 (3)	O2—C13—H13A	111.4 (15)
C3—C2—H2	124.2 (15)	C14—C13—H13A	110.9 (16)
C1—C2—H2	119.0 (15)	O2—C13—H13B	112.0 (14)
C2—C3—C4	121.4 (3)	C14—C13—H13B	111.1 (14)
C2—C3—H3	122.5 (19)	H13A—C13—H13B	104 (2)
C4—C3—H3	116.0 (19)	C13—C14—C15	113.5 (2)
C5—C4—C3	120.6 (3)	C13—C14—H14B	108.3 (15)
C5—C4—H4	119.8 (15)	C15—C14—H14B	111.2 (15)
C3—C4—H4	119.6 (15)	C13—C14—H14A	108.4 (16)
C6—C5—C4	119.6 (3)	C15—C14—H14A	109.8 (16)
C6—C5—H5	117.8 (17)	H14B—C14—H14A	105 (2)
C4—C5—H5	122.6 (17)	C16—C15—C14	112.6 (2)
C5—C6—C1	118.1 (3)	C16—C15—H15A	110.3 (16)
C5—C6—C7	136.4 (3)	C14—C15—H15A	107.9 (15)
C1—C6—C7	105.4 (2)	C16—C15—H15B	109.5 (15)
C8—C7—C12	119.6 (2)	C14—C15—H15B	108.6 (16)
C8—C7—C6	134.6 (2)	H15A—C15—H15B	108 (2)
C12—C7—C6	105.8 (2)	C15—C16—C17	112.8 (3)
C9—C8—C7	117.8 (3)	C15—C16—H16B	109.9 (16)
C9—C8—H8	122.2 (16)	C17—-C16—H16B	109.1 (16)
C7—C8—H8	120.0 (16)	C15—C16—H16A	108.9 (15)
C8—C9—O2	124.2 (2)	C17—C16—H16A	112.5 (15)
C8—C9—C10	121.8 (3)	H16B—C16—H16A	103 (2)
O2—C9—C10	113.9 (2)	C16—C17—H17A	115.1 (18)
C11—C10—C9	120.3 (3)	C16—C17—H17C	110 (2)
C11—C10—H10	119.5 (17)	H17A—C17—H17C	108 (3)
C9—C10—H10	120.1 (17)	C16—C17—H17B	110 (2)
C12—C11—C10	117.5 (3)	H17A—C17—H17B	106 (3)
C12—C11—H11	123.5 (15)	H17C—C17—H17B	106 (3)
